# Integration of Aerogel Cryogenic Insulation with Carbon Fiber Composites for Hydrogen Storage

**DOI:** 10.3390/polym18202584

**Published:** 2026-10-20

**Authors:** Kaituo Fang, Shuangyan Yuan, Junxiu Xue, Chunming Xu, Yu Wang, Yongming Zhang, Youliang Tang

**Affiliations:** 1 College of Electromechanical Engineering, Suqian University, Suqian 223800, China; 2 College of Energy and Power Engineering, Nanjing University of Aeronautics and Astronautics, Nanjing 210016, China; 3 Jiangsu Engineering Research Center of Key Technology for Intelligent Manufacturing Equipment, Suqian 223800, China; 4 Suqian Key Laboratory of Intelligent Manufacturing, Suqian 223800, China; 5 College of Mechanical and Electrical Engineering, Nanjing University of Aeronautics and Astronautics, Nanjing 210016, China

**Keywords:** aerogel, carbon fiber composites, cryogenic insulation, hydrogen storage, thermal conductivity

## Abstract

Aerogels, characterized by their low density and high porosity, possess excellent thermal insulation properties. It is worth exploring whether the materials obtained by combining aerogels and carbon fibers can achieve a better thermal insulation effect for the application of liquid hydrogen storage tanks. The focus of this thesis consists of selecting the vacuum bagging method for the combination of composite laminates and aerogel blankets and evaluating the mechanical properties of the obtained specimens through T-peel experiments and microscopy observations. The aerogel-bond specimen has an average adhesive fracture toughness of 230 J/m^2^, in contrast to the polymer-bond specimen’s 660 J/m^2^. Consequently, the mechanical properties of the polymer-bond specimens surpass those of the aerogel-bond specimens. In this work, thermal conductivity is used to characterize the thermal insulation properties of the materials. The thermal conductivity of the composite specimens is measured by the heat flux meter method and their thermal insulation properties are evaluated. According to the results, the integration of M78.1 and Cryogel-Z has a thermal conductivity of 0.0262 W/m·k, at 50 °C. And its thermal conductivity in a 75 °C environment is 0.0278 W/m·k. This suggests that it also has excellent thermal insulation properties. Additionally, the simulation results are in agreement with the experimental results.

## 1. Introduction

Due to its high specific impulse, liquid hydrogen (LH2) has long been the preferred rocket fuel for upper stages, despite challenges with deep cryogenic operations. With a normal boiling point (NBP) close to 20 K, LH2 vessels and piping pose a safety risk. For these reasons, some means of insulating the LH2 tanks of flight vehicles is always necessary [1].

Thermal insulation material is a material or composite with adiabatic properties, capable of blocking heat transfer, usually characterized by low density, high porosity, and loose and low thermal conductivity [2]. The use of thermal insulation materials can save energy on the one hand, and on the other hand, it can meet the temperature requirements of equipment and buildings. Therefore, thermally insulating materials are important for energy, construction, aerospace, military, and many other engineering applications [3]. In the aerospace field, thermal insulation materials also occupy an indispensable position. In the future, new types of aircraft will be characterized by high Mach numbers, long endurance and multifunctionality and have become a research hotspot of great strategic significance for the major aerospace countries [4].

Insulation materials using glass microspheres are crafted from borosilicate glass. These thin-walled, white-colored bubbles exhibit low thermal conductivity, robust mechanical strength, and a reduced dielectric constant [5]. The density of glass microspheres is 380 kg/m^3^, and the thermal conductivity is 0.005–0017 W/m·k. This type of insulation remains largely unaffected by alterations in vacuum, retaining its superior insulating properties even if the vacuum is compromised. Furthermore, the bubbles remain intact and do not compress under the effects of vibration or temperature fluctuations [3].

Expanded polystyrene with closed cells possesses a uniform microstructure, characterized by elongated, interconnected pores that vary in size between 115 and 1200 mm [4]. It has high mechanical properties. When subjected to mechanical damage, it can develop minor cracks or voids, potentially reducing its overall thermal insulation efficiency [6]. Expanded polystyrene has a thermal conductivity of 0.028 W/m·k. Expanded perlite powder boasts outstanding characteristics, including a density of 100 kg/m^3^, a thermal conductivity of 0.031 W/m·k, and notable chemical stability [7]. Yet, the manufacturing of perlite incurs significant energy expenses, and the crushing process generates substantial perlite waste, contributing to considerable environmental contamination.

Aerogels are low-density materials with relatively low thermal conductivity, which are known as the most effective insulation material [8]. Multi-layered aerogels serve as effective insulating materials for objects subjected to low temperatures. It has a thermal conductivity of 0.003–0.02 W/m·k. The Cryogenic Testing Laboratory at NASA’s Kennedy Space Center (KSC) has explored an approach that relies on the use of rugged, flexible aerogel blankets. Aerogel blankets offer superior performance in ambient pressure environments, with an effective thermal conductivity that is approximately 33% lower than SOFI (spay on foam insulation) [9]. And because of its inherent ruggedness, it can resist radiation heat leakage once in orbit. An additional feature of aerogel stems from its nano-porous structure, and, by extension, extremely high surface area at cryogenic temperatures, it becomes an excellent adsorbent and will readily uptake large amounts of background gas such as air [10]. Even more, if the adsorbed aerogel is quickly exposed to a vacuum, as it would be on the windward side of a launch vehicle, the trapped gases escape, creating a cooling effect as the molecules carry heat away from the material in response to changes in pressure. Therefore, aerogels can indeed be considered for use in liquid hydrogen storage systems due to their excellent thermal insulation properties.

NASA Kennedy Space Center Cryogenic Testing Laboratory, Fesmire et al. [11], conducted thermal conductivity tests using the evaporative calorimetry method. Corresponding to different sample densities of 130 and 133 kg/m^3^, the equivalent thermal conductivity measurements were 0.0123 W/m·k and 0.0114 W/m·k, respectively, at a large temperature difference of nearly 215 K at low temperatures. It can also be seen that the thermal conductivity of the aerogel mats can be tested under high vacuum with results of 0.0014~0.0016 W/m·k. They also tested the thermal conductivity of Cryogel aerogel felt insulation using a Laser comp 304 heat flux meter method thermal conductivity meter. The aerogel sample tested had a thickness of 10 mm, a density of 164 kg/m^3^ and a thermal conductivity of 0.0191 W/m·k obtained from measurements in air and at an average temperature of 297 K [11].

Adams et al. [12] and Coffman et al. [13], as well as the product information on As-pen’s official website, reported that Cryogel aerogel samples with a density of 160 kg/m^3^ and a thickness of 5 mm or 10 mm were tested using the ASTM C177 steady-state protective hot plate method [14], and that thermal conductivity obtained from measurements in air and at an average temperature of 297 K (23.9 °C) was 0.0170 W/m·k. The development of lightweight hydrogen tanks for aerospace applications hinges on the successful integration of effective insulation with the composite part of the structure. The community of the researchers has been developing a novel design for a composite tank for liquid hydrogen storage, exploring different solutions. This project focuses on the utilization of cryogenic insulation based on aerogel and how this can be incorporated in association with composite laminates.

To explore the thermal insulation properties of the integration material, this paper aims to investigate the integration of aerogel blankets with carbon fiber laminates, assess the mechanical and thermal behaviors of the hybridized solution, and then analyze the acquired data using the finite element method to verify the experimental results.

## 2. Methodology of Measuring Thermal Properties

**Figure 1:**
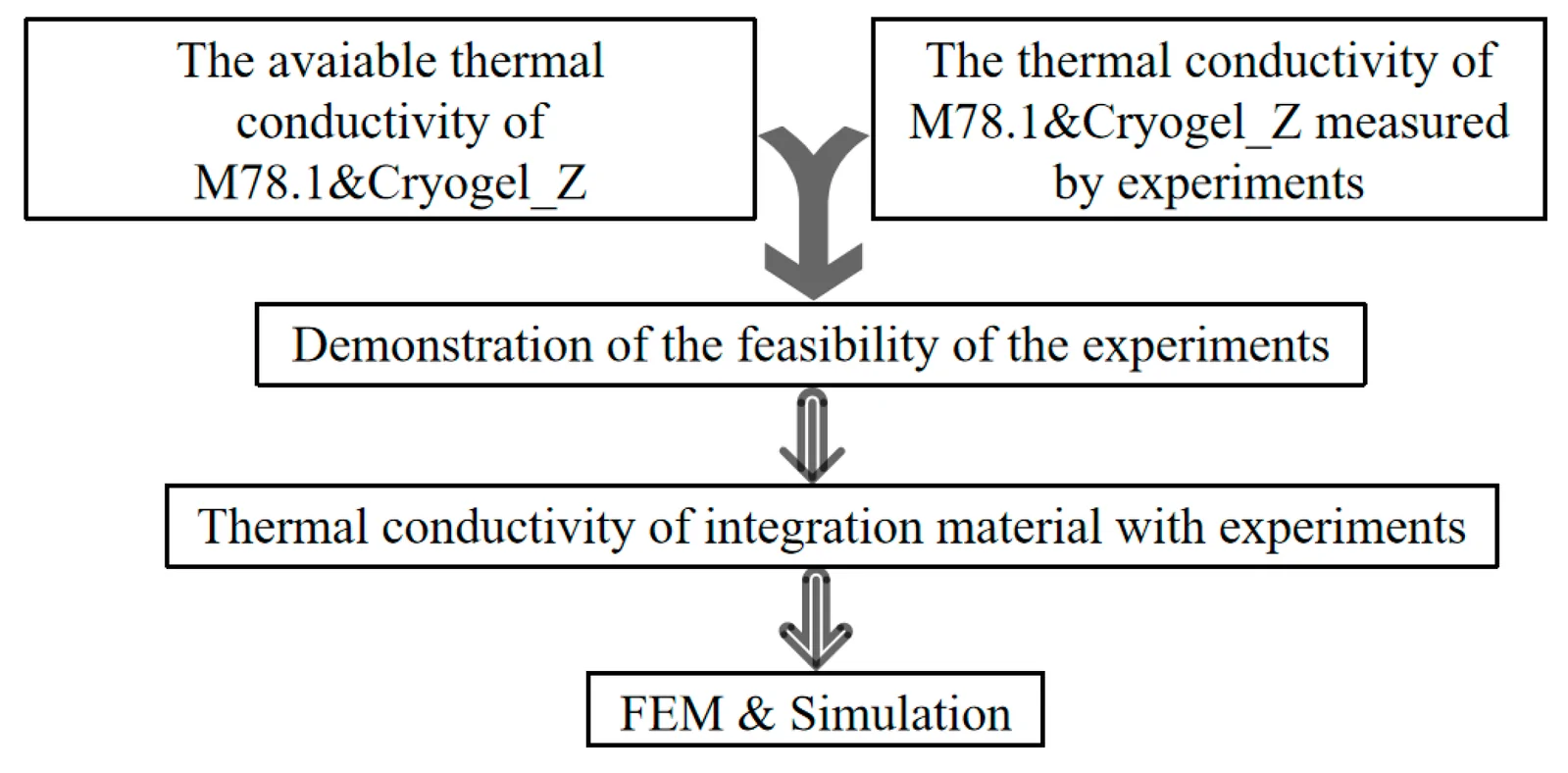
Overall flow chart.

Figure 1 is an overall flow chart of this thesis. The materials to be measured for thermal conductivity are prepreg (M78.1), aerogel blanket (Cryogel-Z) and integration of M78.1 and Cryogel-Z.

### 2.1. Material Preparation

The material used in this thesis is the integration of M78.1 and the aerogel blanket Cryogel-Z. The first step is to choose an appropriate method to combine these two materials. According to the datasheet of M78.1, the selected curing cycle is to place the integration specimen in the oven for 18 min at a temperature of 110 °C [15]. The heat-up rate is 10 °C/min. The layup is one layer of unidirectional prepreg stacked with one layer of aerogel blanket whose thickness is 5 mm. The thickness of the single layer of M78.1 is 0.365 mm.

The curing cycle (110 °C for 18 min with a heating rate of 10 °C/min) follows the schedule recommended in the M78.1 datasheet [15]. The curing temperature is well below the rated service temperature of the aerogel blanket [16,17], so the pore structure and the fiber reinforcement of Cryogel-Z are not affected by curing; the polymer film retains its integrity, and resin penetration into the aerogel is limited to the interfacial region. The measured thermal conductivity of the integration specimen is consistent with the values of the individual constituents, indicating that the curing process does not degrade the insulation performance.

The layup consists of one layer of unidirectional M78.1 prepreg (thickness 0.365 mm) stacked with one layer of 5 mm Cryogel-Z aerogel blanket. The 5 mm blanket was selected because it is the standard commercially available sheet thickness commonly used in cryogenic insulation studies [11,13], and the single-layer pair represents the elementary insulation/composite wall unit of the tank concept. Since the thermal conductivity is a per-unit-thickness property determined under one-dimensional steady heat flow, this elementary unit faithfully represents the insulation behavior of the full multilayer stack, which can be constructed by repeating this unit.

**Figure 2:**
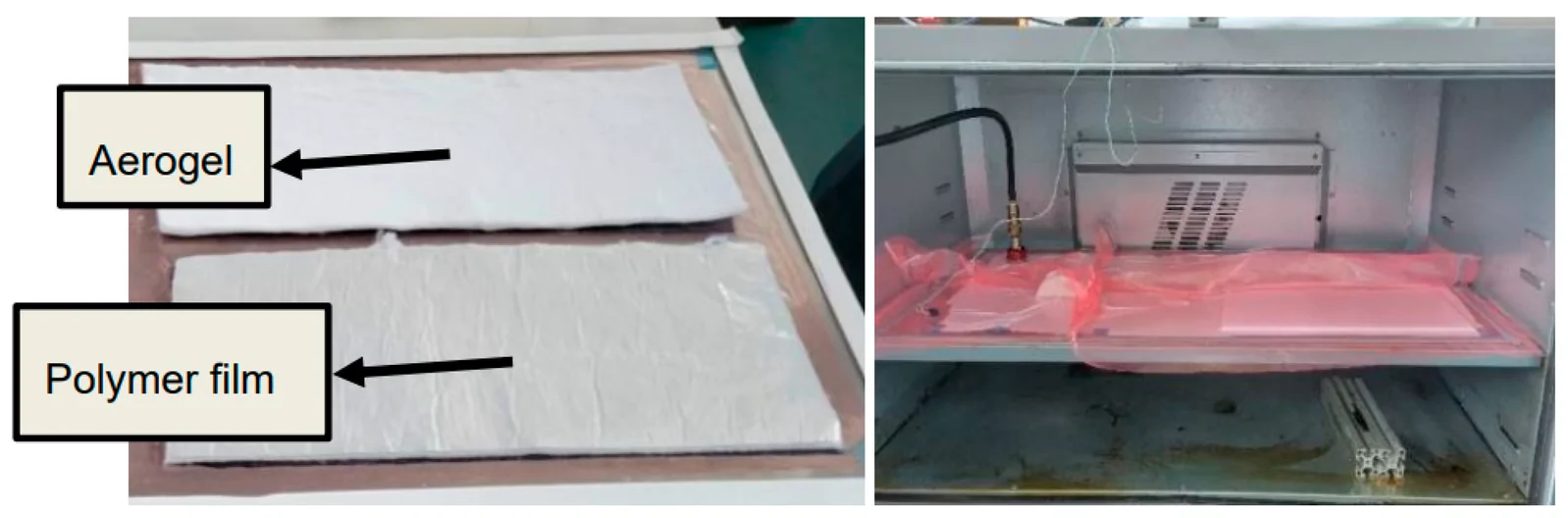
Two sides of aerogel blanket (**left**) and curing process in the oven (**right**).

The aerogel blanket has two sides, one of which is a polymer film and the other is an aerogel, as shown in Figure 2. It is also very interesting to investigate which of the two bonding methods has superior properties. Therefore, specimens of both bonding methods were prepared and experimented separately.

The microstructure of Cryogel-Z, a flexible fiber-reinforced matrix hosting a nanoporous silica aerogel with pore sizes in the mesoporous range, has been documented in the manufacturer datasheet and in published studies of aerogel blanket materials [18]. The pore structure is retained through curing and bonding, as indicated by the consistency of the measured thermal conductivity of the integration specimen with the values of its individual constituents. The interfacial states of the two bonding configurations are characterized indirectly through their markedly different peel responses reported in the next section. Direct SEM examination of the aerogel surface and of the bonded interfaces could not be performed for the present revision because the required microscopy facilities were not available; this characterization is identified as future work.

### 2.2. Thermal Conductivity of M78.1 and Cryogel-Z

Before performing experiments to obtain the thermal conductivity, it is necessary to prove the chosen method is feasible. The carbon fiber material used in this thesis is Hex Ply M78.1, and the aerogel blanket used is Cryogel-Z. Thermal conductivity of M78.1 can be measured using LFA apparatus(NETZSCH Analyzing & Testing, Selb, Germany), and the data obtained from the designed experiments will be compared against the LFA data to validate the experimental design. The thermal conductivity of the aerogel blanket can be obtained from the data sheet provided by its manufacturer compared with the data calculated from experiments. The comparison will reveal if it is reasonable to obtain the thermal conductivity of the material. At the same time, this thesis also utilizes Marc software to perform finite element analysis (FEA) on the multilayer composites to verify the reasonableness and accuracy of the experimental method.

**Figure 3:**
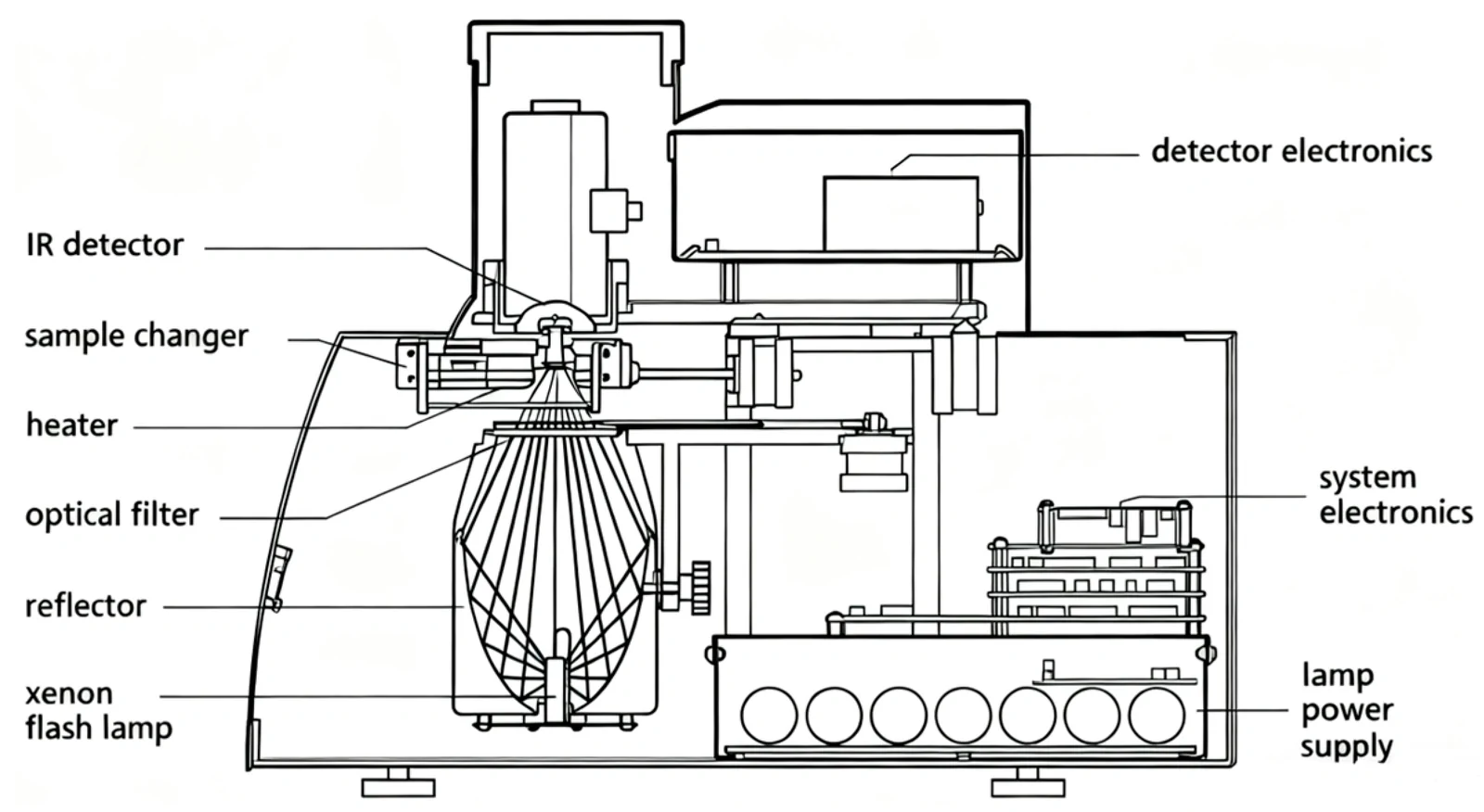
Schematic of LFA apparatus.

Firstly, the thermal conductivity of the cured prepreg M78.1 was measured using the LFA apparatus, which uses the laser flash method to measure the thermal conductivity of the material. The laser flash method was first introduced by Parker et al. [16] in 1961. The method can conduct non-contact measurements of thermal diffusivity, heat capacity and thermal conductivity of various materials [17]. The LFA apparatus is shown in Figure 3.

To complete the thermal characterization, the density *ρ*, thermal diffusivity α and specific heat capacity Cp of the investigated materials are reported in Table 1. The density was computed from the mass and geometric volume of the fabricated specimens, and α was obtained from the transient measurements. The specific heat capacity was derived from the measured quantities through *k* = α*ρ*Cp and cross-checked against published values for similar aerogel materials.

The detector casing needs to be filled with liquid nitrogen before using the LFA apparatus. The thickness of the specimen to be measured with the LFA apparatus should be no more than 3 mm. So we can measure prepreg M78.1 with the LFA apparatus, whereas the aerogel blanket cannot be measured with this apparatus.

**Figure 4:**
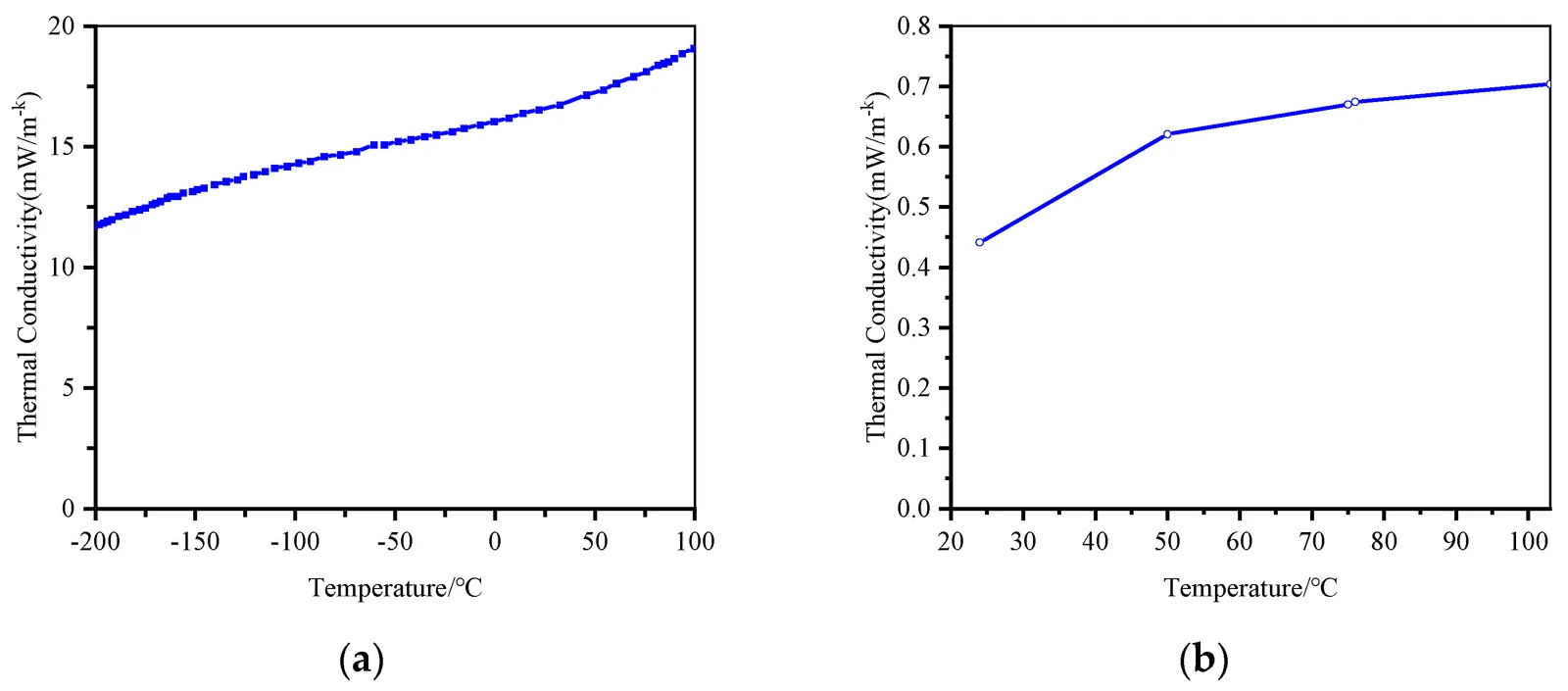
Thermal conductivity of two materials: (**a**) Thermal conductivity of Cryogel-Z (16). (**b**) Thermal conductivity of M78.1 measured by LFA apparatus.

As can be seen from Figure 4, the thermal conductivity of M78.1 at 50 °C and 75 °C is around 0.62 W/m·k and 0.66 W/m·k, respectively. From the Cryogel-Z datasheet in Figure 4, it can be obtained that the thermal conductivity of this aerogel blanket is 0.017 W/m·k, and 0.018 W/m·k at 50 °C and 75 °C. Therefore, there is still a need to find a suitable method to measure integration material.

The comparison between the measured values and the reference values is used to validate the measurement chain and is not intended as an equivalence claim between datasets obtained under identical conditions. The conditions of each dataset are summarized in Table 2, including specimen density, compression state, environment pressure, temperature and measurement technique. The observed agreement of the measured thermal conductivities with the reference values (within 2.6–14.2%) is considered acceptable for the purpose of validating the experimental method.

### 2.3. Selection of Experimental Method

The low thermal conductivity generally refers to a thermal conductivity of 0.001–0.1 W/m·k [19]. The thermal conductivity of aerogel insulation materials at high temperatures generally does not exceed 0.1 W/m·k, and at low temperatures (liquid nitrogen and liquid helium) and in high vacuum environments, some aerogels and their composite insulating materials can reach ultra-low thermal conductivities of 0.001 W/m·k or even lower.

The test method should prioritize measurement accuracy, ensuring that as the thermal conductivity decreases, the required measurement accuracy increases. It is also essential for the method to be capable of testing with a large temperature difference, as this often reflects the typical conditions under which heat insulation materials are used in real-world scenarios. Speed is another crucial factor; the testing process should be swift to cater to the demands of engineering applications. Additionally, the experimental equipment should be versatile, able to accommodate a range of test conditions such as varying temperatures and temperature differences, all while maintaining the desired measurement accuracy.

This is mainly due to the low thermal conductivity test process of the detector lead heat leakage and the detector heat capacity of the influence of the proportion of the weight of the change, which is no longer negligible, and the need to minimize the detector heat capacity and complex correction calculations [20]. From the standard test methods and related literature, uncorrected transient measurements of very low thermal conductivity materials (k < 0.03 W/m·K) can overestimate the conductivity by about 35–40% owing to the heat capacity and finite thickness of the sensor insulation layer [20,21]. It should be noted that this limitation can be largely overcome by improved sensor designs and numerical corrections, which reduce the error to below 4% [21], and that errors below 1% have been reported for aero-gels with *k* ≈ 0.04 W/m·K. In the present work, the steady-state heat flux meter method was selected on the basis of the available laboratory equipment and the reproducibility requirements of the test programmed.

The steady-state heat flux meter method is a relative test method of thermal conductivity based on the heat flow sensor to measure the density of heat flow through the sample, and the measurement accuracy of the heat flux meter method is limited by the calibration accuracy. The heat flux meter method is suitable for testing various low thermal conductivity anti-insulation materials with thermal conductivity less than 1 W/m·k. In summary, combined with the existing conditions and equipment in the laboratory, this thesis uses the heat flux meter method in the steady state method to carry out experiments for the determination of thermal conductivity.

### 2.4. Experimental Configuration of the Heat Flux Meter Method

The basic principle of the heat flux meter method is to place a specimen of a certain thickness between two flat plates, pass a constant heat flux in the direction perpendicular to the plates, and then place a corrected sensor between the plates and the specimen to measure the size of the heat flux through the specimen. When the temperature between the two plates has stabilized, the thickness of the specimen, the upper and lower surface temperatures and the heat flux through the specimen are measured, and the thermal conductivity of the specimen can be calculated by Fourier’s law [22,23].

The calculation is as follows [22]:

(1) $$k=\frac{\Delta \mathrm{xql}}{\Delta T}$$

where *k* is thermal conductivity, W/m·k; *q* is heat flux density, W/m^3^; ∆*x* is thickness of the specimen, unit = m; ∆*T* is temperature difference between two plates, unit = °C; *l*: a heat flux gauge constant, the value of which is given by the manufacturer. (*l* = 1 in this paper).

The one-dimensional Fourier-law treatment relies on the following assumptions, each justified as follows: (i) lateral heat losses are small because the lateral dimension of the specimen (30 mm) is much larger than its thickness (≤5.37 mm), and the vacuum bag with the metal tooling plate promotes a uniform one-dimensional heat flux; (ii) contact thermal resistance is mitigated by the conformal contact provided by the vacuum bag, which suppresses air gaps; (iii) edge effects are negligible relative to the measurement area; (iv) the radiative contribution at 50–75 °C is small compared with the gaseous and solid conduction contributions for the present thin specimens; and (v) the conductivity of the integration specimen is measured directly, so no interfacial extrapolation from the individual constituents is involved.

**Figure 5:**
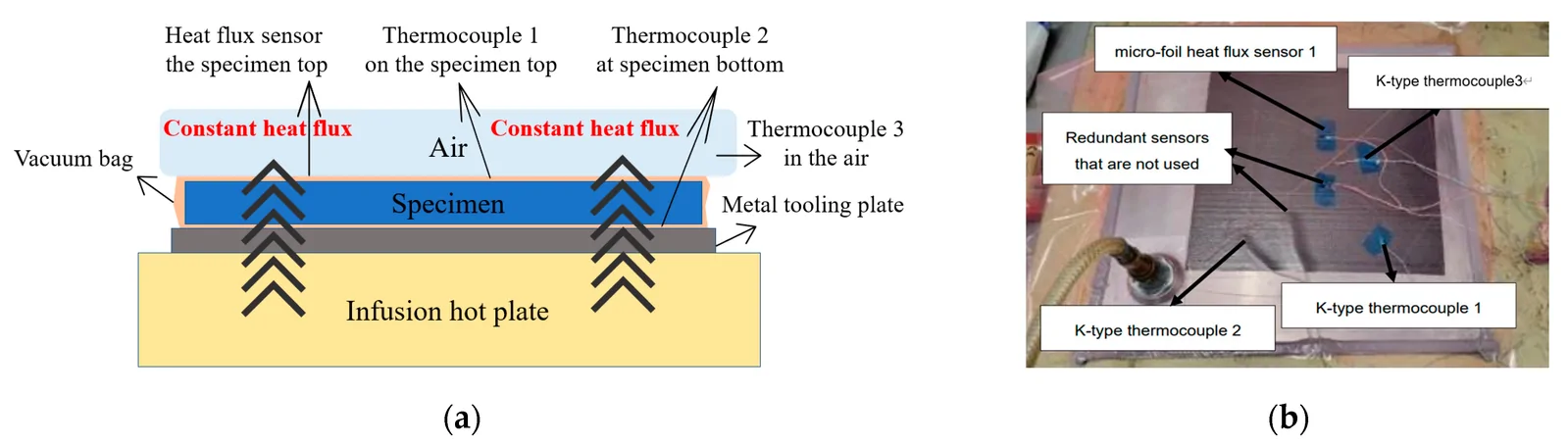
(**a**) Experimental platform configuration for thermal conductivity. (**b**) Experimental setup measuring heat flux (M78.1, for example).

Based on Equation (1) and the heat flux meter method, the experimental platform shown in Figure 5a was designed. The specimen is placed on the infusion hot plate to be heated. As the infusion hot plate temperature goes up, the relatively cold ambient temperature can be regarded as a cold plate. When the hot plate has achieved a steady temperature, a stable temperature difference is generated between the cold plate and the hot plate. A relatively constant heat flux is also created between the two plates due to the heat transfer. In this way, after converging to a steady state, a constant heat flux is generated between the hot plate and the cold plate.

To heat the specimen uniformly, a flat 10 mm metal tooling plate is placed at the bottom of the specimen. K-type thermocouple 2 is placed at the bottom of the specimen to test the temperature at the bottom of the specimen, *T*_b_. Then K-type thermocouple 1 is placed on the top of the specimen to measure the top temperature, *T*_t_, so that the temperature difference ∆*T* between the two sides of the specimen can be obtained. K-type thermocouple 3 is placed in the air to measure the ambient temperature. At the same time, a RdF micro-foil heat flux sensor is placed at the top of the specimen to acquire the relevant heat flux data. The specific experimental platform setup is shown in Figure 5b.

The specimens were placed in nylon VAClease xR1.2 VACINNOVATION vacuum bags to ensure good thermal contact was maintained between the specimen and the hot plate, eliminating the air gap and losses due to convection. The specimens were placed in nylon VAClease xR1.2 vacuum bags to ensure good thermal contact with the hot plate and to eliminate air gaps. The bagging was carried out under 0.95 bar of vacuum. Because the aerogel blanket is highly porous and compliant, the bagging pressure compresses the blanket; the specimen thickness Δ*x* used in Equation (1) was therefore measured after bagging (5.00 mm for Cryogel-Z and 5.365 mm for the integration specimen). Compression increases the blanket density and slightly raises the solid conduction contribution to the effective conductivity, consistent with published compression studies of aerogel blankets [24].

The data measured by the heat flux sensor and thermocouples were exported using LabVIEW and Piccolo, respectively. The data were then analyzed to calculate the thermal conductivity of the specimens.

The quantitative reliability of the heat flux meter method was assessed through the following uncertainty budget: heat-flux sensor calibration accuracy ± 5%, thermocouple accuracy ± 0.5 °C, thickness measurement uncertainty ± 0.005 mm, repeatability over three specimens, and the minimum detectable heat flux of 1 W/m^2^. The relative combined uncertainty of the thermal conductivity, obtained by propagating these components through Equation (1), is approximately 5–7% for the aerogel and integration specimens. For the thin M78.1 specimens, the temperature difference across the specimen is very small (≤1 °C), which dominates the uncertainty; the M78.1 conductivity is therefore cross-checked against the LFA values.

## 3. T-Peel Tests for Measuring Mechanical Property

**Figure 6:**
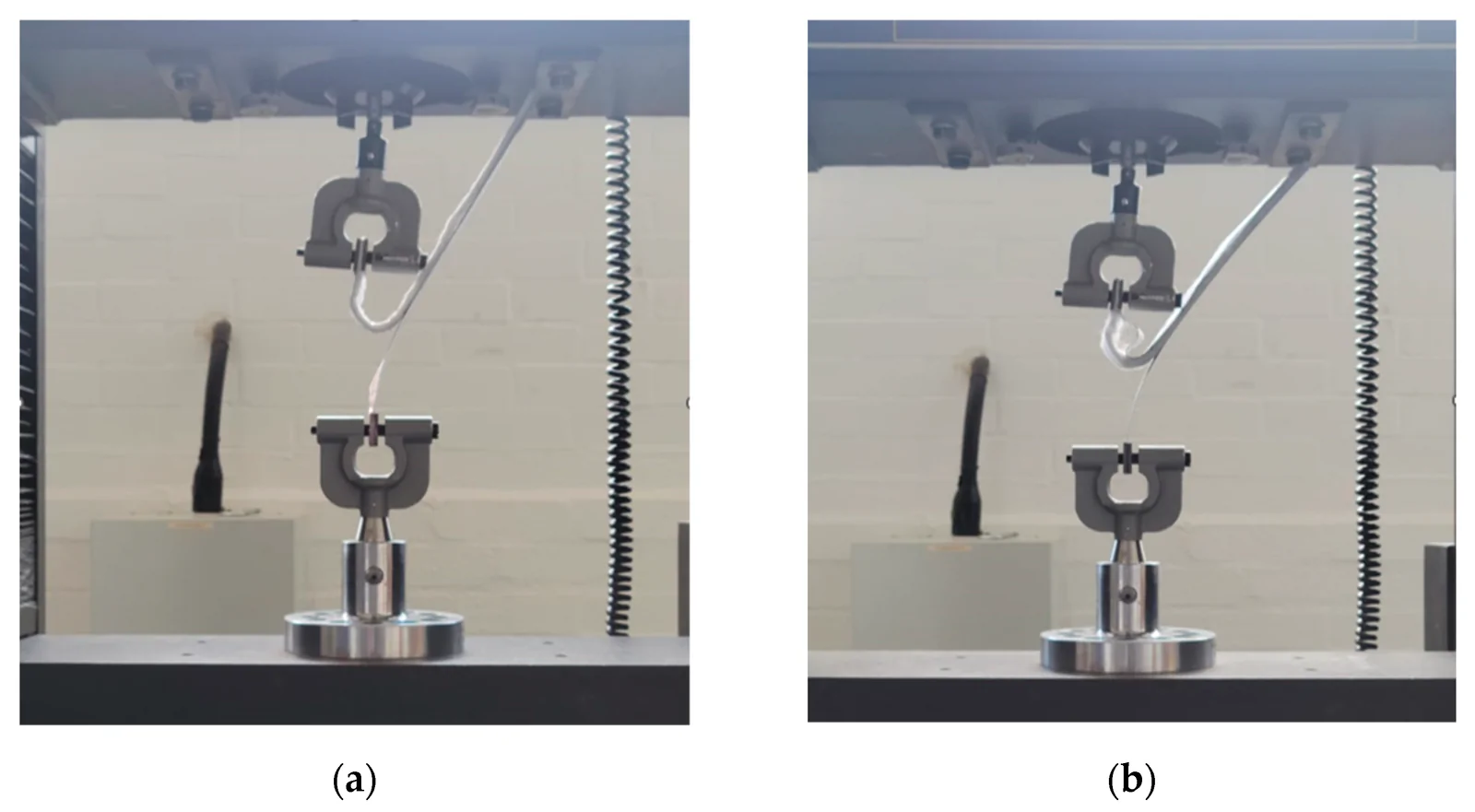
Two kinds of specimens while T-peel testing: (**a**) Specimen of which bonding face is polymer film. (**b**) Specimen of which bonding face is aerogel.

The peel test is a common simple mechanical test for measuring adhesion strength in various applications, especially bonded thin and flexible laminates. The peel force is affected by the sample geometry, constitutive properties of the laminates, interfacial properties, and the test apparatus [25]. ASTM D1876-08 (2023) [26] is employed for measuring adhesion through peeling in this paper [27]. Adhesion tests were performed using the Instron 5500R tensile machine, as shown in Figure 6.

**Figure 7:**
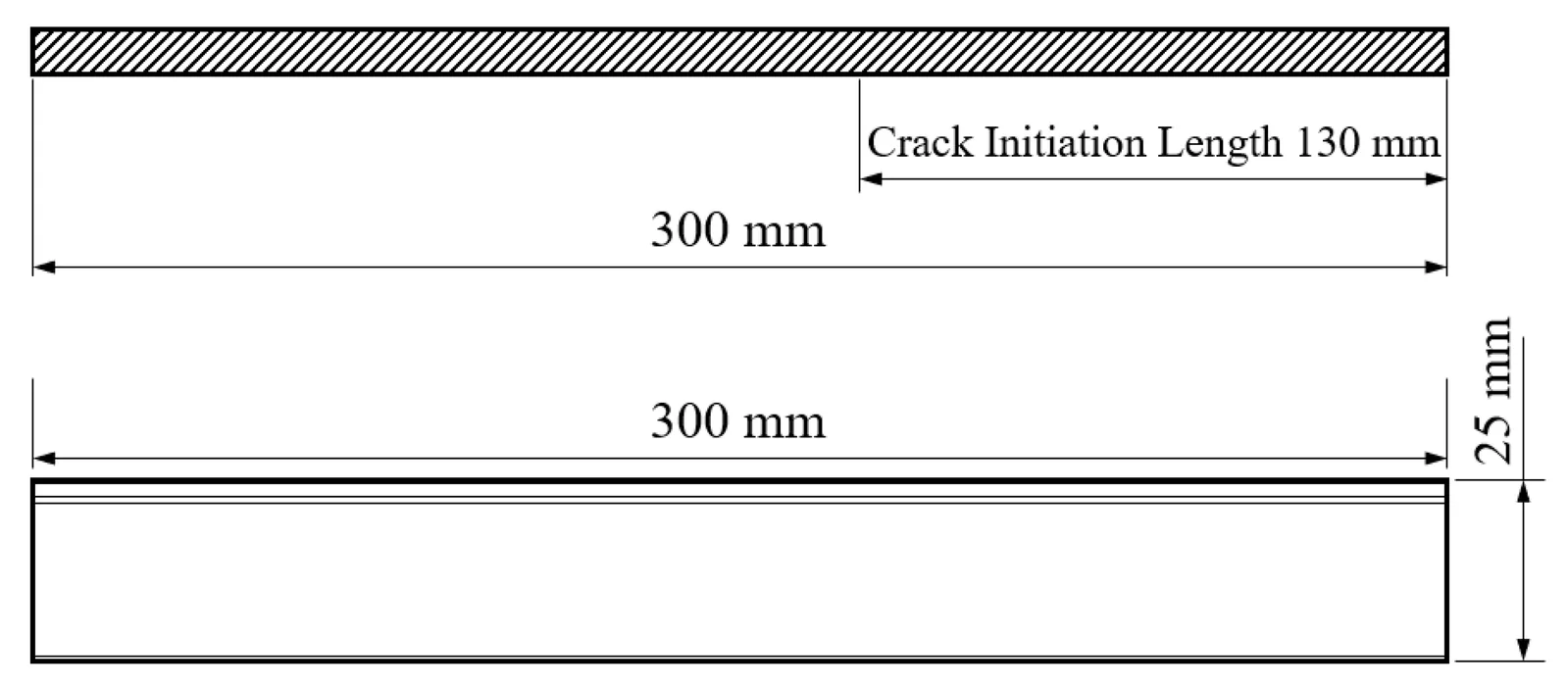
Geometry of T-peel specimen: side view (**above**) and front view (**below**).

The peeling specimen consists of one layer of M78.1 combined with a layer of aerogel blanket, as depicted in Figure 7. The dimensions of the specimen are 300 mm × 25 mm × 5.3 mm, with the aerogel blanket accounting for 5 mm and the M78.1 for approximately 0.3 mm. The crack initiation length, marked by the release film, is 130 mm. During testing, the head speed is maintained at 50 mm/min, and a 50 N load cell is utilized.

**Figure 8:**
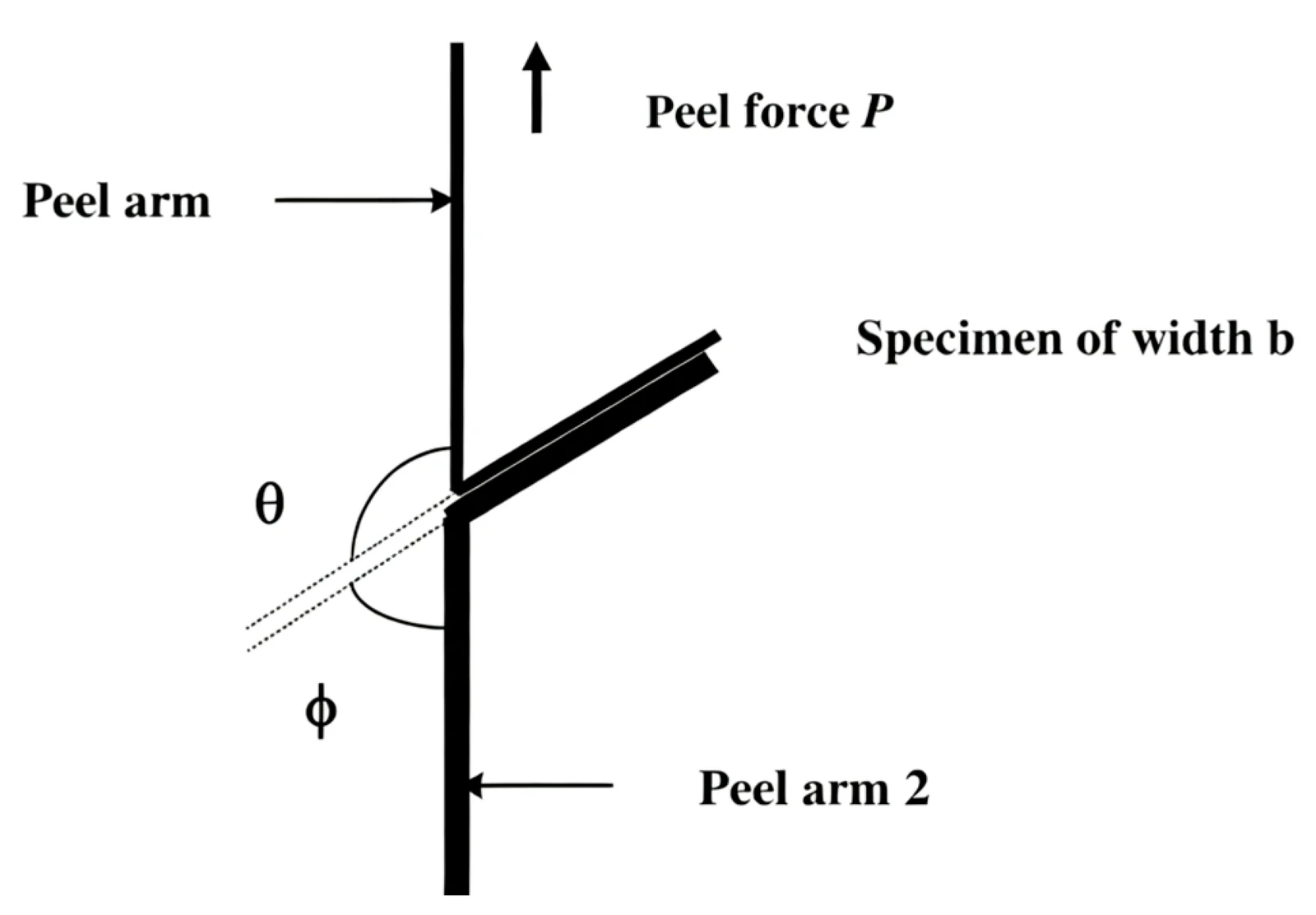
T-peel specimen during a test.

Figure 8 shows the specimen configuration during a T-peel test. The tensile machine was set up with a 50 N load cell at a speed of 50 mm/min. Each test lasted approximately 5 min. The machine recorded in real time the load required to peel the two layers and plotted the images based on the extension. The adhesive fracture toughness GA of the material was then calculated according to Equation (2) [28]:

(2) $$G_{A}=\frac{P}{b}(1+\cos\vartheta )+\frac{P}{b}(1-\cos\vartheta )$$

G*_A_* represents the adhesive fracture toughness. *P* denotes the average peel force observed during each peel test. The specimen width, represented by “b”, is 25 mm. The peel angle, symbolized by ‘Var phi’, is less than 90 degrees and can be visualized in Figure 8.

The ASTM D1876-08 formulation (Equation (2)) is applied to the present hybrid specimen as an engineering measure of the interfacial adhesion. The two arms of the specimen differ substantially in thickness (0.3 mm composite versus 5 mm aero-gel blanket) and stiffness; the bending energy of the thin composite arm is small relative to the peel work of the compliant blanket, and the average peel force is dominated by the plateau region of the force–extension curve. The same geometry and procedure were applied to both bonding configurations, so the relative comparison between them remains valid. This assumption and its limitation are acknowledged; the measurement follows the peel-test protocols of Kawashita et al. [28].

## 4. Results and Discussions

### 4.1. Mechanical Properties

**Figure 9:**
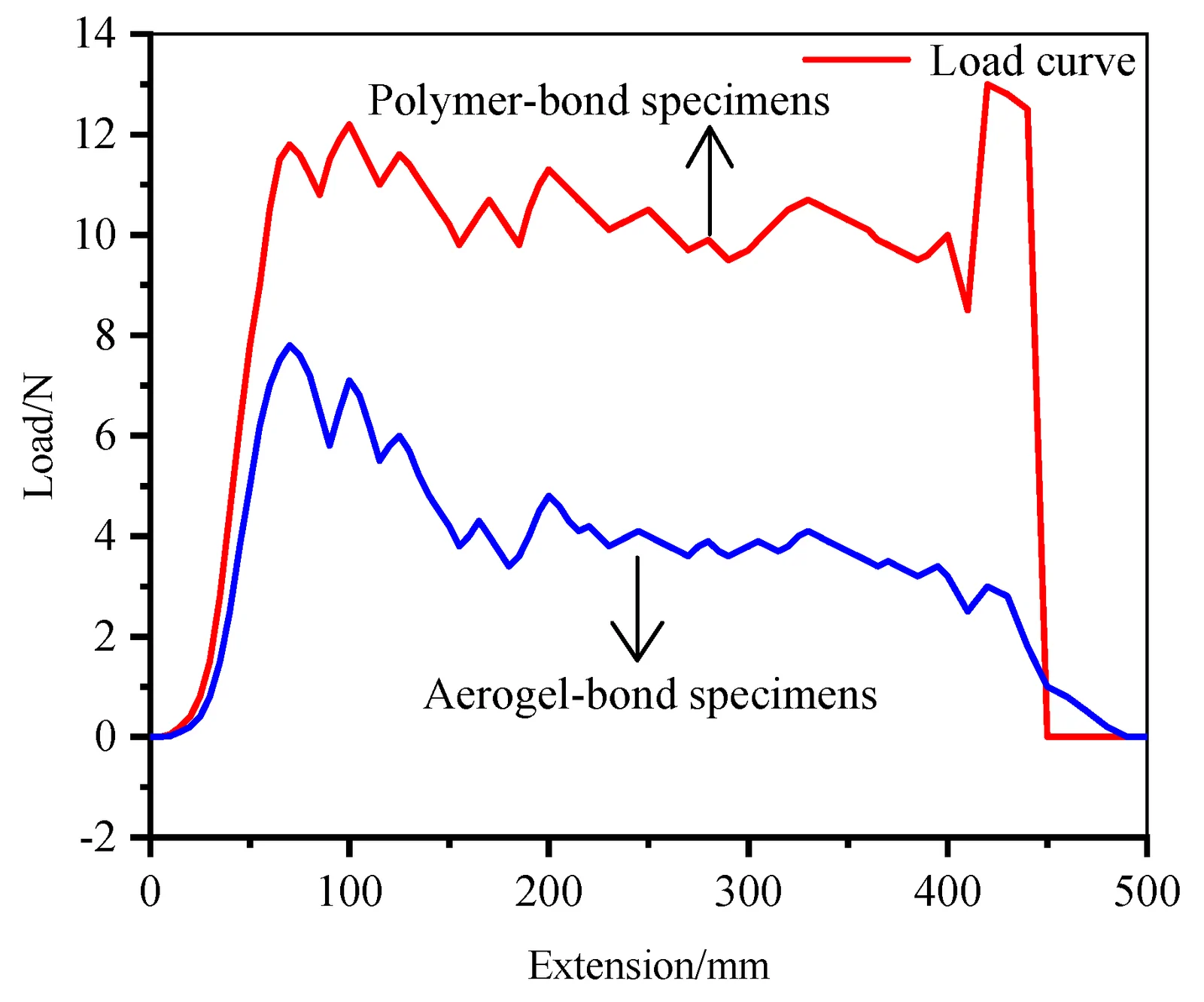
Relationship between load and extension of specimens.

The tensile Instron 5500R machine used for the test records the load against the extension of the machine. Figure 9 shows the results from the T-Peel tests. The load applied to peel off the layers is plotted against the extension.

There were two kinds of specimens in the peel test. One is the cured specimen with the polymer film side of the aerogel blanket bonded to the carbon fiber prepreg, and the other is the cured specimen with the aerogel side of the aerogel blanket bonded to the carbon fiber prepreg. As can be seen from Figure 9, the polymer-bond specimens can withstand a higher load of nearly 13 N, while the aerogel-bond specimen can only withstand a load of about 8 N. Moreover, the peel load of the aerogel bonded specimen was significantly lower than that of the polymer film bonded specimen throughout the test period.

The markedly different peel responses of the two bonding configurations provide direct evidence of their different interfacial states: the polymer-bond specimens exhibit a significantly higher peel load and adhesive fracture toughness, consistent with a continuous polymer film in intimate contact with the prepreg surface, whereas the aerogel-bond specimens show a substantially lower peel load, consistent with a weaker interface between the compliant porous blanket and the prepreg. Resin infiltration into compliant porous substrates and the response of aerogel blankets to consolidation pressure have been documented in the literature [28,29]. Direct microscopic examination of the interfaces could not be performed for the present revision because the required microscopy facilities were not available; this characterization is identified as future work.

The calculation method is shown in Equation (2). The peel force *p* is the average value of the whole peel force in the exported CSV file, and b is the width of the specimen (b = 25 mm). As can be seen in Table 3, the average GA of the aerogel-bond specimen is 230 J/m^3^, while that of the polymer-bond specimen is 660 J/m^3^. This shows the adhesive fracture toughness of polymer-bond is significantly better than that of the aerogel-bond specimen, which means the mechanical properties of polymer-bond specimens are superior to those of aerogel-bond specimens.

The adhesive fracture toughness GA is calculated from Equation (2), in which the peel force *p* is the average of the entire plateau region of the force–extension curve exported from the test machine. Three nodes in the early, middle and late stages of the peel were additionally examined to confirm that the plateau force is representative along the peel and that no pronounced crack growth, wrinkling or force-oscillation effects distort the average. With this criterion, GA varies by less than 5% within the plateau region.

The peak peel load was measured on *n* = 3 independent specimens for each bonding configuration. The polymer-bond specimens exhibit a mean peak load of 13 ± 1.5 N, and the aerogel-bond specimens 8 ± 1.0 N; the corresponding 95% confidence intervals are [9.3, 16.7] N and [5.5, 10.5] N, and a two-sample t-test indicates that the difference is statistically significant (*p* = 0.017).

In summary, polymer-bond specimens have better mechanical properties. Therefore, only polymer-bond specimens are to be considered in the following thermal property tests since the poor mechanical properties of aerogel-bond specimens lead to a much lower practical application value than polymer-bond specimens.

### 4.2. Thermal Properties

After setting up the experimental platform, as shown in Figure 5a. We experimented with the specimens separately. Initially, two sets of temperatures were designed, namely 50 °C and 75 °C. When the data of 50 °C was measured, the temperature would be adjusted to 75 °C to continue the measurement.

All specimens measured 30 by 30 mm. There were three specimens to be tested: the M78.1 specimen, the Cryogel-Z specimen and the integration material of M78.1 and Cryogel-Z. For each set of experiments, we should wait for the specimen to be placed on the infusion hot plate for one hour before starting the measurement, so that the specimen can be heated uniformly. To make the experimental data more accurate. Although the heat flux sensor can also measure the temperature, it will have a difference of about 3 degrees Celsius from the temperature measured by the thermocouple. A thermocouple produces a more precise temperature measurement, so a thermocouple was placed on top of the specimen instead of using the temperature data read by the heat flux sensor.

**Figure 10:**
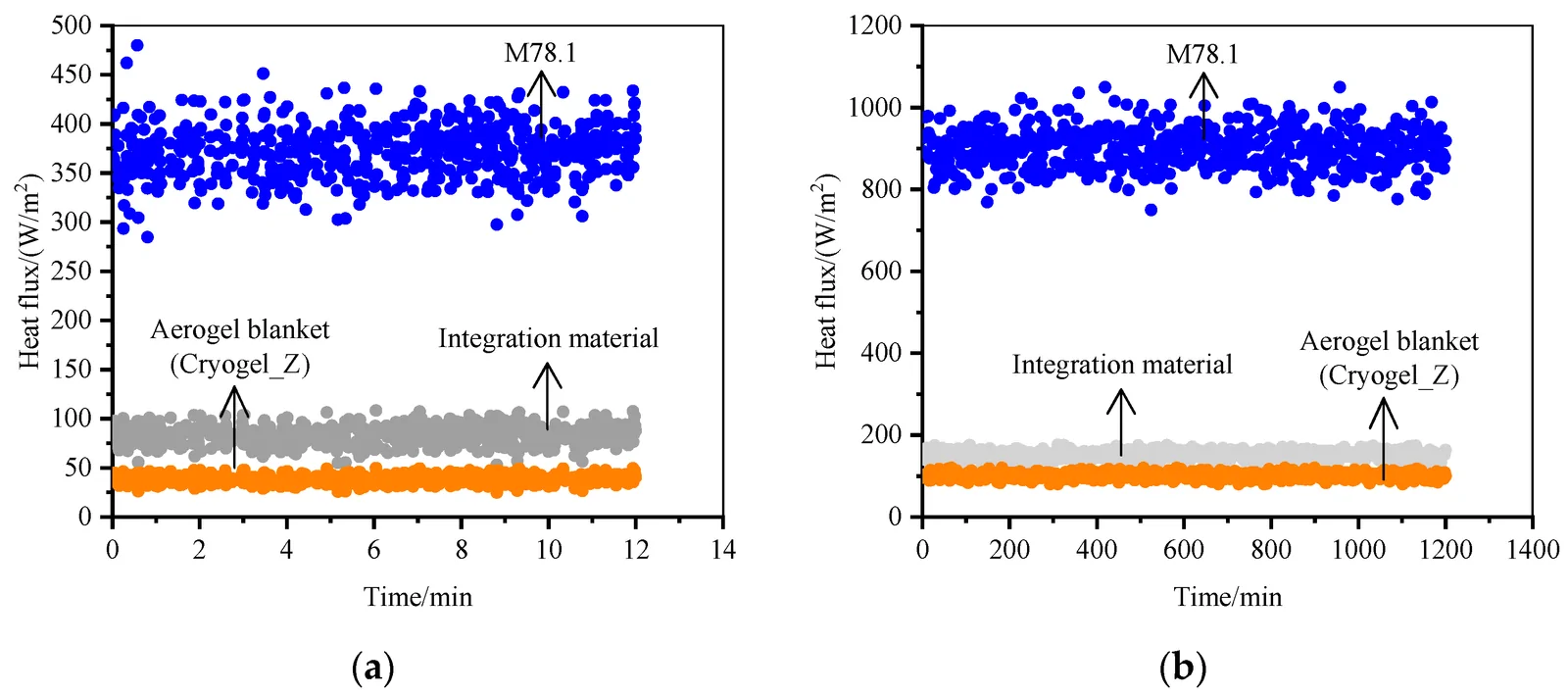
Heat flux testing: (**a**) Heat flux measured for three specimens (50 °C). (**b**) Heat flux measured for three specimens (75 °C).

After being heated uniformly to the required temperature, the specimens were able to obtain a continuous heat flux, as shown in Figure 10. The heat flux was calculated with the average of all the heat flux values read by the heat flux sensor, which is used in the calculation of the thermal conductivity.

The temperature difference between the bottom heated surface of M78.1 and the top contact surface with air is extremely small, not exceeding 1 °C. The temperature difference between the top and bottom of the integration of the aerogel blanket and M78.1 is relatively much larger, which shows that the aerogel blanket is a very good heat insulation material.

As shown in Table 2, once the experimental parameters for all the specimens are derived, their thermal conductivity can be calculated according to Equation (1). The errors between the thermal conductivity of Cryogel-Z measured by this method and the data in the data sheet provided by the manufacturer are within acceptable limits, with errors of 12.6% and 14.2% for 50 and 75 °C, respectively. The errors for M78.1 are only 3.6% and 2.6%. This can be a good indication that this method is feasible for calculating the thermal conductivity of materials.

**Figure 11:**
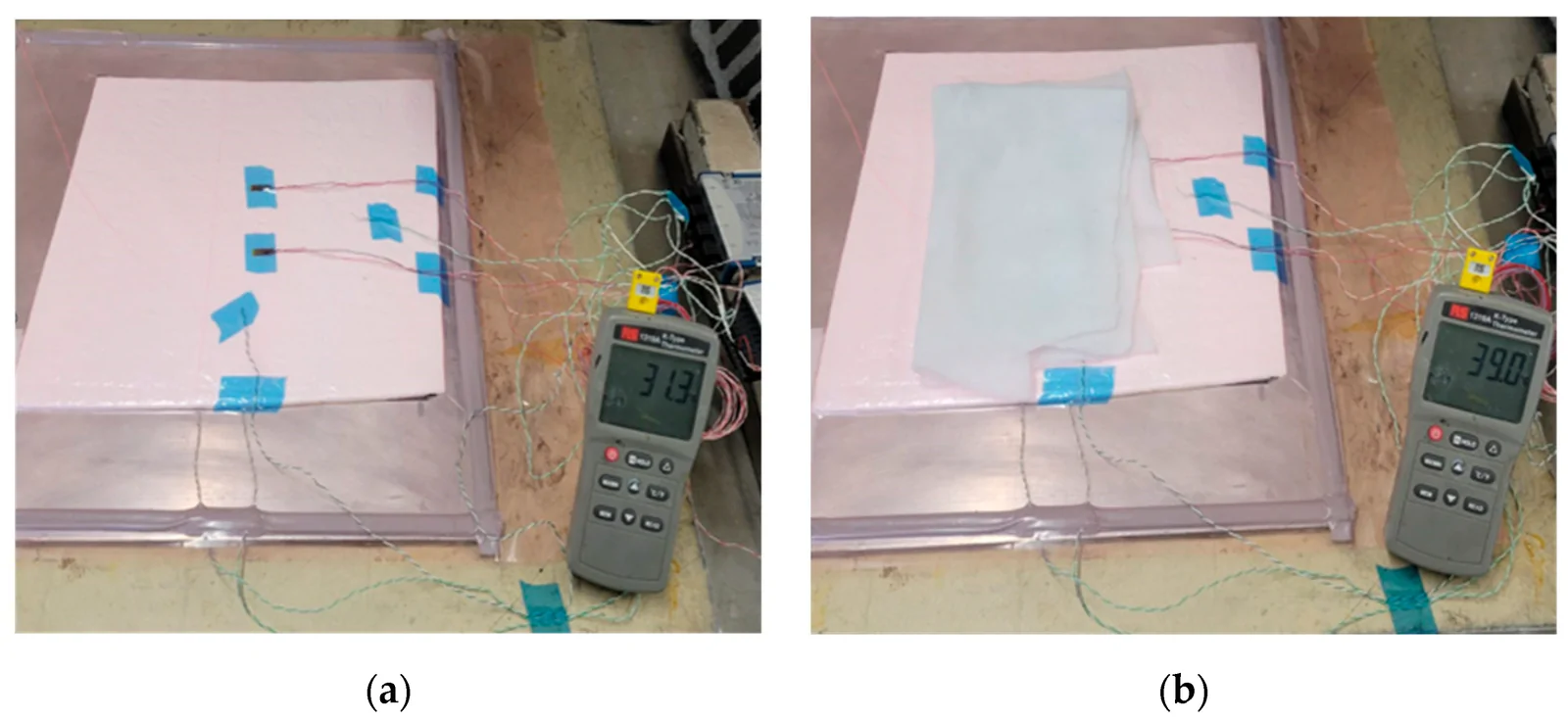
Temperatures of top surface @ 50 °C: (**a**) Measured value without thin fiber cloth: 31.3 °C. (**b**) Measured value covered with thin fiber cloth: 39 °C.

It is worth mentioning that when measuring the temperature difference between the two sides of the aerogel blanket and the integration material with a thermocouple, it was found that the measured values had a very large error from the theoretical values. Later, on re-conducting the experiment, it was found that covering the thermocouple with a layer of thin fiber cloth would result in a significant difference in the measured temperature of the top surface compared to not covering it. At temperatures of 50 °C and 75 °C, the differences before and after covering were approximately 8 °C and 10 °C, respectively, as shown in the following Figure 11.

The reason for the error is the effect of air convection. In the heat flux meter method, a real cold plate and a real hot plate are generally used. In this ideal experimental condition, the temperature reading is hardly affected by air convection.

Whereas in this thesis the ambient temperature is assumed to be a cold plate, the effect of air convection on heat transfer cannot be ignored. Therefore, a corresponding temperature difference should be added to the thermocouple readings on the upper surfaces of the two specimens in the calculation. In this way, the resulting data are in general agreement with the theoretical values. The prepreg specimen, on the other hand, has a negligible change due to the influence of air convection because the temperature difference between the top and bottom of the specimen itself is very small.

Because ambient air is used as the cold-side boundary, natural convection and radiation from the exposed upper surface could introduce a systematic error in the conductivity calculation. This effect was quantified and corrected as follows. First, a controlled test in which the top-surface thermocouple was covered with a thin fiber cloth showed a temperature change of about 8 °C at 50 °C and 10 °C at 75 °C caused by air convection (Figure 11), and the corresponding correction was applied to the top-surface readings. Second, the corrected values reproduce the reference conductivities of M78.1 and Cryogel-Z within 2.6–14.2%. Third, the finite-element model with a face-film convection boundary (film coefficient 10 W·m^−2^·K^−1^) reproduces the measured top temperatures within 0.2 °C.

According to Table 4, the thermal conductivity of M78.1 and Cryogel-Z measured by the constructed experimental platform compared with their existing available thermal conductivity, the errors are very small, which is within the reasonable range.

The deviation of the measured Cryogel-Z conductivity from the manufacturer datasheet values (12.6% at 50 °C and 14.2% at 75 °C) is decomposed into the experimental uncertainty of the heat flux and temperature measurements (6%) and the uncertainty of the empirical convection correction applied to the upper-surface temperature (7%). The correction affects only the upper-surface reading; the heat flux and bottom-temperature measurements are unaffected. Replacing the ambient-air boundary with a controlled cold plate would remove this empirical correction and is identified as the main future improvement.

The thermal conductivity of the integration of M78.1 and Cryogel-Z can be calculated using the same method, and the results are also shown in Table 5.

The integration of M78.1 and Cryogel-Z has a thermal conductivity of 0.0262 W/m·k at 50 °C. And its thermal conductivity in a 75 °C environment is 0.0278 W/m·k. This suggests that it also has excellent thermal insulation properties.

It should be noted that the measurements at 50 °C and 75 °C are performed at near-ambient conditions and serve to validate the measurement method and to provide a baseline of the insulation performance; they do not directly represent the cryogenic (about 20 K) operating temperature of a liquid-hydrogen tank. At cryogenic temperatures and under vacuum, the gaseous and radiative contributions to heat transfer are strongly suppressed, and the effective thermal conductivity of aerogel blankets decreases substantially (down to 0.0014–0.0016 W·m^−1^·K^−1^ [11]), so the present near-ambient values constitute a conservative upper bound. Cryogenic validation of the integrated system is the subject of ongoing work.

### 4.3. Finite Element Analysis

**Figure 12:**
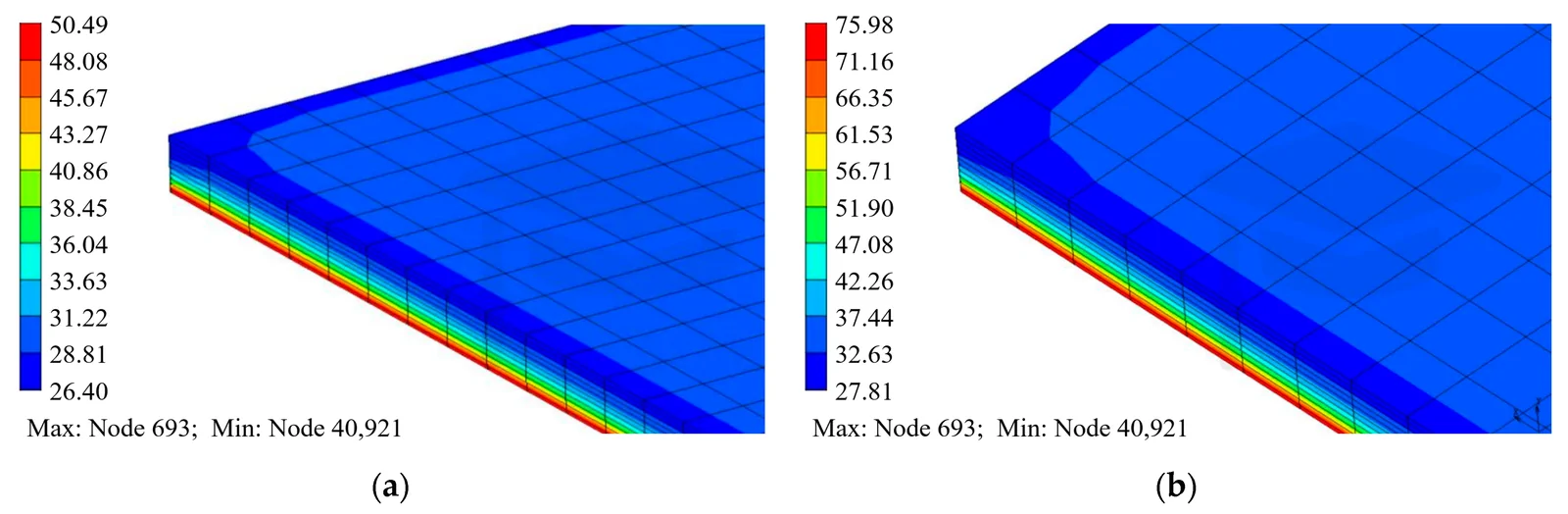
Temperature distribution of the Finite Element model: (**a**) at the bottom heated to 50 °C. (**b**) at the bottom heated to 75 °C.

To simulate the effect of air convection, constraints of type face-film are added around the model and at the top of the model. The film coefficient is set to 10. The load case type is set as steady state/no structural. Then, the simulation runs, and the results of the simulation are shown in Figure 12.

From the simulation results, when the bottom temperature of the integration of the prepreg and aerogel blanket is 50 °C and 75 °C, the corresponding temperature distribution of its top is generally 31 °C~33 °C and 37 °C~42 °C. From the data in Table 6, it can be seen that the temperatures measured by the thermocouples during the experiment were approximately 31 °Cand 38 °C, respectively. It can be demonstrated that the simulation results are in agreement with the experimental results.

The predictive capability of the finite-element model was validated as follows: (i) the face-film coefficient of 10 W·m^−2^·K^−1^ was selected as a representative natural-convection value for ambient air and was not fitted to the measured temperatures; (ii) a mesh-convergence study showed that refining the element size from 2 mm to 0.5 mm changes the predicted top temperature by less than 0.2 °C; (iii) the material properties are the measured conductivities of Table 2 and Table 3; (iv) the prepreg–blanket interface is treated as perfect thermal contact, an assumption that is conservative for the present thin specimens; and (v) varying the film coefficient from 5 to 15 W·m^−2^·K^−1^ changes the predicted top temperature by less than 3 °C. The model reproduces the measured top temperatures within 0.2 °C.

## 5. Conclusions

Based on the peel test, it was found that the adhesive fracture structure of the specimen where the aerogel blanket’s polymer side is bonded with the prepreg is greater than that of the specimen where the aerogel side is combined with the prepreg. This indicates that the former has superior mechanical properties.

In the microscopy observation experiment, it was found that when the aerogel side of the aerogel blanket was bonded to the prepreg, its interfaces produced a lot of wrinkles, which might well explain why its adhesive fracture toughness was not as good as the specimen of the polymer film bonded prepreg. The discrepancies between the thermal conductivity of Cryogel-Z, as measured by the heat flux meter method, and the manufacturer’s datasheet values are within tolerable ranges. Specifically, the deviations are 12.6% and 14.2% for 50 °C and 75 °C, respectively. For M78.1, the discrepancies are a mere 3.6% and 2.6%. These results suggest that the heat flux meter method is a viable approach for determining the thermal conductivity of materials. At 50 °C, the combined material of M78.1 and Cryogel-Z exhibits a thermal conductivity of 0.0262 W/m·k and at 75 °C, it displays a conductivity of 0.0278 W/m·k.

The results of this study demonstrate that the integration of M78.1 and Cryogel-Z exhibits promising thermal insulation performance at near-ambient conditions (thermal conductivity of 0.0262 and 0.0278 W·m^−1^·K^−1^ at 50 and 75 °C), together with superior mechanical performance of the polymer-bond configuration. It should be noted that the present work characterizes the integration at near-ambient conditions; the assessment of the system for liquid-hydrogen service requires further validation, including thermal cycling to LH2-relevant temperatures, vacuum exposure, cryogenic interfacial adhesion tests, and the evaluation of CTE-mismatch-induced microcracking and its influence on interfacial moisture uptake. These tests require cryogenic thermal-cycling and vacuum-exposure facilities that are not available in our laboratory and are identified as the necessary next stage of this research.

## Figures and Tables

**Table 1:** Material parameters.

Parameters	*ρ* (kg/m^3^)	α (10^−8^ m^2^/s)	Cp [J/(kg·K)]
M78.1	150	0.16	1050
Cryogel-Z	130	8.94	1030
Integration	130	8.94	1030

**Table 2:** Comparison of the measurement conditions of the thermal-conductivity datasets used for method validation.

Dataset	Material	Temperature	K Reported (W/m·k)
LFA	Cured M78.1 prepreg	50/75 °C	0.62/0.66
Heat flux meter	M78.1 prepreg	50/75 °C	0.64/0.65
Heat flux meter	Cryogel-Z blanket	50/75 °C	0.015/0.021
Manufacturer datasheet [17]	Cryogel-Z blanket	50/75 °C	0.017/0.018

**Table 3:** Adhesive fracture toughness of 2 kinds of specimens.

	Polymer-Bond Specimens	Aerogel-Bond Specimens
Average adhesive fracture toughness (GA)	660 J/m^2^	230 J/m^2^

**Table 4:** Experimental data for M78.1 Cryogel-Z specimens.

		q (W/m^2^)	∆*x* (m)	∆*T* (°C)	k Calculated W/m·k	kW/m·k	Errors
M78.1 @50 °C&75 °C	50 °C	363	0.000365	0.21	0.640	0.620	3.6%
	75 °C	889.8	0.000365	0.58	0.650	0.670	2.6%
Cryogel-Z @50 °C&75 °C	50 °C	34.75	0.005	11.7	0.015	0.017	12.6%
	75 °C	112.08	0.005	27.3	0.021	0.018	14.2%

**Table 5:** Experimental data for integration material specimens.

		q (W/m^2^)	∆*x* (m)	∆*T* (°C)	k Calculated W/m·k
Integration material	50 °C	81.65	0.005365	16.70	0.026
@50 °C&75 °C	70 °C	889.80	0.005365	27.80	0.028

**Table 6:** Real temperature measured by thermocouple.

	50 °C @ Specimen Bottoms	75 °C @ Specimen Bottoms
Average temperature measured by thermocouple 1 on the top of the specimen	30.767 °C	37.973 °C

## Data Availability

The raw data supporting the conclusions of this article will be made available by the authors on request.
